# Effect of Gonadotropin Types and Indications on Homologous Intrauterine Insemination Success: A Study from 1251 Cycles and a Review of the Literature

**DOI:** 10.1155/2017/3512784

**Published:** 2017-12-13

**Authors:** Rosalie Cabry-Goubet, Florence Scheffler, Naima Belhadri-Mansouri, Stephanie Belloc, Emmanuelle Lourdel, Aviva Devaux, Hickmat Chahine, Jacques De Mouzon, Henri Copin, Moncef Benkhalifa

**Affiliations:** ^1^ART and Reproductive Biology, University Hospital and School of Medicine, Picardie University Jules Verne, CHU Sud, 80054 Amiens, France; ^2^PERITOX-INERIS Laboratory, CURS, Picardie University Jules Verne, CHU Sud, 80054 Amiens, France; ^3^Laboratoire Lavergne, ACCOLAB, 75016 Paris, France; ^4^Unilabs France et ForteBio, 1 rue Mozart, 92200 Clichy La Garenne, France

## Abstract

**Objective:**

To evaluate the IUI success factors relative to controlled ovarian stimulation (COS) and infertility type, this retrospective cohort study included 1251 couples undergoing homologous IUI.

**Results:**

We achieved 13% clinical pregnancies and 11% live births. COS and infertility type do not have significant effect on IUI clinical outcomes with unstable intervention of various couples' parameters, including the female age, the IUI attempt rank, and the sperm quality.

**Conclusion:**

Further, the COS used seemed a weak predictor for IUI success; therefore, the indications need more discussion, especially in unexplained infertility cases involving various factors. Indeed, the fourth IUI attempt, the female age over 40 years, and the total motile sperm count <5 × 10^6^ were critical in decreasing the positive clinical outcomes of IUI. Those parameter cut-offs necessitate a larger analysis to give infertile couples more chances through IUI before carrying out other ART techniques.

## 1. Introduction

Infertility, defined as the inability to achieve a desired live-birth after 1 year of unprotected and regular sexual intercourse, concerns 10–15% of couples [1–3] or more [4].

Intrauterine insemination (IUI) is often the first-line procedure in assisted reproductive technologies (ART) in France due to its simplicity and low cost, but it is also less effective [5]. Although IUI technique is widely used to treat infertile couples with mild male factor infertility, anovulation, endometriosis, unexplained infertility, and other infertility causes [6–9], the limited IUI success rate can be affected by several factors with little consensus [5]. Among them, the female's age, the male's sperm quality, the IUI attempt rank, the infertility type, and the used gonadotropin for controlled ovarian stimulation (COS) are considered the most predictive factors of IUI clinical outcomes [10–16].

However, IUI combined with COS may increase the cumulative pregnancy rate [17], while in UK clinics, as reported recently by Kim et al. [18], 98% of carried IUI are using COS, the most commonly used medications of which are gonadotropins (95%) [5, 18]. On the other hand, some studies were more focused on the infertility type and the effectiveness of COS in IUI, especially for couples with endometriosis [19–21], male factor, unexplained infertility, and more [15, 16].

Furthermore, many studies have evaluated the predictive factors of IUI clinical outcomes, including the female's age, the infertility duration, the rank of the attempt, and the sperm parameters [10–16]. However, few studies have compared the pregnancy rates based on the used COS or on the infertility type [5, 22–25].

We took the opportunity of having a large sample of IUI cycles in a single centre to try to analyse the effect of stimulation protocols and of the infertility origin on the results, taking in account the main confounding variables (female age, IUI attempt rank, and sperm quality) on the clinical outcome.

## 2. Materials and Methods

### 2.1. Patient Selection

We selected in our retrospective observational cohort study all couples attending our reproductive medical centre to obtain homologous IUI program with COS between January 2007 and August 2014, with the following inclusion criteria: women with a failure to conceive after ≥12 months of unprotected and regular intercourse, aged 20–44 years, with normal ovulation reserve (basal follicle-stimulating hormone (FSH) level < 10 IU/l and estradiol (E2) level < 30 ng/mL); their partners had to have a total motile sperm (TMS) count of >1 × 10^6^. The exclusion criteria were: TMS ≤ 1 × 10^6^; sperm donation; seropositivity for human immunodeficiency virus (HIV) for any couple member; inseminations performed in a natural cycle or with clomiphene citrate (CC).

### 2.2. IUI Protocol

All couples had undergone a standard infertility evaluation, which included medical history, physical examination, and assessment of tubal patency by either hysterosalpingography or laparoscopy and hormonal analysis on cycle day 3. A transvaginal ultrasound scan was performed on the second day of the cycle. On the same day, ovarian stimulation was carried out with recombinant FSH (follitropin *α*; rFSH; Gonal-F, Merck Serono, France, or follitropin *β*; Puregon, MSD, France), urinary FSH (urofollitropin, Fostimon, France), or hMG (menotropin, Menopur, France) at a starting dose of 75 IU/day from the second day of the cycle.

Ovarian response and endometrial thickness were monitored by transvaginal ultrasonography starting on day 6 of stimulation and then on alternate days; the gonadotropin dose was adjusted according to the ovarian response and the patient's characteristics. When at least one mature follicle reached a diameter >17 mm and E2 level > 150 pmol/mL, the recombinant human chorionic gonadotropin (hCG, Ovitrelle, Merck Serono, France) was administered, and endometrial thickness was evaluated.

A single IUI was performed 36 h after hCG injection using a soft catheter (classic Frydman catheter; Laboratoire CCD, Paris, France) or a hard catheter (SET TDT, International Laboratory CDD). The semen samples used for insemination were processed within 1 hour of ejaculation by density gradient centrifugation, followed by washing with a culture medium after determining the TMS and semen analysis according to the WHO criteria [26].

### 2.3. Outcome Variable

The main clinical outcome measures were clinical pregnancy and live-birth rates per cycle. Clinical pregnancy was defined as the evidence of pregnancy by ultrasound examination of the gestational sac at weeks 5–7.

### 2.4. Statistical Analysis

The stimulation protocols were divided into 4 categories according to the gonadotropin used for COS: rFSH/Gonal-F, rFSH/Puregon, uFSH/Fostimon, and hMG/Menopur.

Infertility type was considered in seven categories: cervical factor, dysovulation, endometriosis, tubal factor, male factor, and unexplained infertility. After statistical analysis of the results, it was necessary to determine the parameter cut-offs to give infertile couples more chances through IUI before carrying out other ART techniques

Groups were compared for all main couples' characteristics and cycle outcomes. Data are presented as mean ± standard deviation (SD) or percentage of the total. Data were analysed with Student's *t*-test for means comparisons or with the chi-squared test for comparison of percentages using Statistical Package, version 9.3 (SAS; Institute Inc., Cary, NC, USA); *p* < 0.05 was used to define significant differences. Then, a multivariate analysis was performed using logistic models (SAS). For this analysis, all the COS involving FSH were regrouped, versus HMG. A power calculation was performed to determine the differences that could be demonstrated from the sample size of the different COS groups constituted from this retrospective cohort study, with a power of 80%, an error risk of 5%, and a bilateral design.

### 2.5. Ethical Standards

The study was approved by the ethics committee of Picardie University Hospital Jules Verne, and all patients signed written informed consent after receiving a detailed description of the study design, protocol, and outcomes. The authors declare that all procedures contributing to this work comply with the ethical standards of the relevant national and institutional committees on human experimentation and with the Helsinki Declaration of 1975, as revised in 2008.

## 3. Results

### 3.1. General Characteristics

The study population characteristics are described in Table 1. The mean female age was 31.6 ± 4.3 years (20–44 years); 21.8% were over 35 years of which 2.8% were aged over 40 years. The mean IUI attempt rank was 2.2 ± 1.3, 39.6% of couples with a first IUI cycle, and 15% with more than 3. Thus, 36% of patients who carried out IUI had unexplained infertility, 22% had a cervical factor, 16% had PCOS, and in 12% a male factor was present. The mean sperm concentration was 76.0 × 10^6^/mL, sperm motility mean was 24%, and TMS mean was 9.4 × 10^6^, but 8%, 21%, and 85% of couples had less than 15 × 10^6^/mL of sperm concentration, 5 × 10^6^ of TMS, and 40% of sperm motility, respectively, which were considered as the limit according to the WHO standards [26]. However, clinical pregnancy and live-birth rates were 13% and 11%, respectively. COS was distributed as follows: rFSH/Gonal-F (*n* = 362); rFSH/Puregon (*n* = 538); uFSH/Fostimon (*n* = 210); and hMG/Menopur (*n* = 141). Infertility type was cervical factor (*n* = 276); dysovulation (*n* = 91); PCOS (*n* = 205); endometriosis (*n* = 35); tubal factor (*n* = 44); male factor (*n* = 153); and unexplained infertility (*n* = 447).

### 3.2. Effect of Controlled Ovarian Stimulation

There was no difference between the four COS groups (Table 2) concerning the couples' characteristics (women's age, semen characteristics, and number of previous cycles). Furthermore, there also was no significant difference in clinical pregnancy rates between the four groups respectively, 12.7%, 13.4%, 13.3%, and 14.2% (*p* = 0.98), for rFSH/Gonal-F, rFSH/Puregon, uFSH/Fostimon, and hMG/Menopur groups, respectively (Table 2). The same was true for the live-birth rate (11.6%, 11.5%, 11.4%, and 13.5%; *p* = 0.99).

Among the couples' characteristics analysed, only the IUI attempt rank was negatively correlated to the clinical outcomes for each group. On the opposite, sperm motility was only positively significantly correlated to live-birth in the 2 rFSH groups. Other couples' parameters—especially female age—did not show a significant correlation with clinical outcomes of IUI whatever COS was used (Table 3).

### 3.3. Effect of Infertility Type

There was no significant difference across infertility types for both clinical pregnancy and live-birth rates (Table 4), even if there were some variations.

Female age was negatively correlated to live-birth for patients with cervical infertility. The IUI attempt rank was negatively correlated to the delivery rate for patients with PCOS, male factor, or unexplained infertility. Sperm concentration was positively correlated to clinical pregnancy and live-birth for couples with male infertility, and the TMS was positively correlated to clinical pregnancy in dysovulation cases (Table 5).

### 3.4. Multivariate Analysis

A multivariate logistic model was applied, including all the potential factors of IUI success (Table 6). The chance of clinical pregnancy was significantly and negatively affected by unexplained infertility, by women's age ≥ 40 years and by a high IUI rank (≥4). On the opposite, chance was increased when the TMS exceeded 5 × 10^6^. Concerning the chance of live-birth, it was only reduced for an high IUI rank (Table 6). The others OR were at the same level as for clinical pregnancy, but not reaching significance. However, a model taking into account age as a continuous variable (not shown) showed a significant impact for delivery (OR = 0.95; 95% CI: 0.91–0.99), but not for pregnancy (OR = 0.97; 0.93–1,01)

The power calculation showed a power of 80% to demonstrate a difference across the COS groups in delivery rates of 10% between groups 1 and 4 and 2 and 4, of 11% between groups 3 and 4, of 8% between groups 2 and 3, of 8% between groups 2 and 4, of 7% between groups 1 and 2, 6% between recombinant FSH and urinary products, and of 9% between FSH and HMG

## 4. Discussion

As a first step in ART, IUI keeps a central place in the management of infertile couples for its simplicity, but it still offers weak effectiveness. Indeed, IUI success is still a subject of controversy, with a clinical pregnancy rate between 8% and 25% [16, 18, 27–31]. Furthermore, based on a recent prospective study in seven French ART centres, the overall live-birth rate was 11% per cycle, varying from 8% to 18% between centres [9]. Similarly, we attained 13% for clinical pregnancy and 11% for live-birth for the 1251 couples who underwent homologous IUI with gonadotropins for COS (Table 1).

Indeed, gonadotropin use had proved its superiority to improve clinical outcomes of IUI compared to other COS protocols, such as CC and letrozole [32–38]. Erdem et al. [36] showed that, for IUI success, rFSH (Gonal-F) was more effective than using CC to reach 28% for clinical pregnancy and 24% of live-birth. Nevertheless, it is still not clear which of the currently available medications is preferable for COS [15, 23, 39–43]. However, several studies compared different types of gonadotropin efficiency (rFSH, uFSH, or hMG) [15, 25, 44–47]. Indeed, in the first part of this work, we compared four gonadotropins for COS in IUI (rFSH/Gonal-F; rFSH/Puregon; uFSH/Fostimon and hMG/Menopur) while rFSH was the most used in 72% of couples (Table 1).

This preference was noticed in other studies [9, 15, 25, 36] without finding any significant improvement on clinical outcomes. Indeed, as demonstrated in our study, there was no significant difference between different protocols used for COS (rFSH/Gonal-F; rFSH/Puregon; uFSH/Fostimon; and hMG/Menopur; Table 2), although, in contrast, some authors pointed to the greater potency of rFSH [22, 48]. However, other studies have reported higher pregnancy rates for hMG [33, 49–53]. Even if our study had 80% power to demonstrate differences in PR of 6% to 11% between 2 groups, according to their size, it is clear that the differences we observed were very low, in favour of a low impact of the 4 used COS regimen on the results. This was less clear for infertility origin because of the very low numbers of some groups. However, the results of the multivariate logistic model confirmed the results observed at the first step analysis, reinforcing their value

Generally, rFSH is commonly used to minimize the possibility of developing ovarian cysts associated with LH contamination and to improve the probability of a more consistent, effective, and efficient ovarian response [22, 48].

Although there was no significant difference between the efficiency of gonadotropins for COS, other COS protocol factors could be involved to improve the clinical outcomes, especially regarding the starting dose and the total doses of treatment as proved by several studies [15, 23–25, 54].

To explain the absence of a significant difference between the four COS groups, we analysed other factors relative to COS protocol (female age, IUI attempt rank, and sperm quality). As expected, our studied population showed its heterogeneity involving multiple factors, which was the reason not to have a real consensus about the efficiency of COS, and this made it harder to really evaluate its impact. The sperm motility significantly affected the live-birth in rFSH groups (Table 3). Furthermore, the IUI attempt rank had a significant negative correlation with clinical outcomes with unequal values between groups (Table 3). Indeed, it is not legitimate to consider the COS as a strong predictive factor of clinical outcomes in IUI, while other factors could not all be controlled

Infertility type has been discussed throughout several studies as a nonnegligible indicator of IUI clinical outcomes [15, 30, 38, 50, 55–59], while the latest National Institute for Health and Care Excellence (NICE) guideline on fertility [59] recommends that IUI should not be routinely offered to people with unexplained infertility, mild endometriosis, or mild male factor infertility who are having regular unprotected sexual intercourse.

For this reason, in the second part of this study, we were more focused on evaluating the infertility type effect on IUI success. As a result, there was no significant difference between clinical outcomes of the different groups based on the infertility type (Table 4). Although unexplained infertility was most couples' indication for IUI (36%) (Table 1), as noticed in the recent report of Monraisin et al. [9] with a value of 39%, the lack of significant difference in clinical outcomes with other IUI indications was not unexpected, while its aetiology kept the multifactorial profile [57] shared with other infertilities. Our results are confirmed by the recent study of [38]. However, some teams report the best pregnancy rates in cervical indications [30, 55] and in anovulation infertilities [15, 50, 56]. Indeed, the pregnancy rate per cycle for patients with anovulation due to PCOS was 13%, which was probably corrected by Controlled Ovarian Hyperstimulation (COH) [15]. On the other hand, endometriosis was considered a bad prognostic factor for IUI success with lower pregnancy (between 6% and 9%) than other IUI indications [20, 50, 60]. Indeed, endometriosis, which is among the most difficult disorders to treat [21], decreased the IUI success rate for mild compared to severe cases (6% of success rate). This fact can argue the limitation of IUI to a maximum of two to three cycles [15, 19, 50, 60, 61]. This fact could explain our weak population size in the endometriosis group with just 35 couples, while the majority of couples were directed to undergo IVF.

Several predictors of success have been widely studied on the COS effect and the infertility type effect. The most discussed effect was the age of the women, with a large debate on its impact on IUI success. Age has been accepted by many authors as a major predictive factor for pregnancy after IUI [29, 30, 60].

The female age was a predictive variable for the live-birth rate but not for clinical pregnancy due to the increased miscarriage rate with age dependence, as can be observed in predictive unadjusted models [9, 57, 62]. The female age became a significant variable predictive for clinical pregnancy and live-birth rate with an adjusted model designed by Van Voorhis et al. [63] and, subsequently, Hansen et al. [57].

In contrast with the aforementioned authors, our results did not show a significant correlation between the women's age and the clinical pregnancy rate (Table 1), which was confirmed by several studies [11, 15, 16, 28, 64, 65]. This is due both to the intervention of other factors used in patients' selection (including ovarian reserve) and to the low numbers of women aged 40 or more.

Nevertheless, the female age impacted the success of IUI. A recent study by Bakas et al. [66] demonstrated a significant negative correlation between the age of the women and the clinical outcome of IUI (*r* = −0.7). Indeed, with the female age cut-off of 40 years, clinical pregnancy was significantly affected (Table 6) as shown throughout several studies, while the pregnancy rate decreased from 13–38% to 4–12% when the women were older than 40 years [30, 60, 67].

The female age impact on IUI success could be masked in our study, because only 21.8% were over 35 years and 2.8% over 40 years. There may be a too low power to show a significant impact of age 40 and more in the multilogistic model, even if OR for this age category was very low (0.17). Moreover, a multilogistic model including age as a continuous variable showed a significant negative impact on the delivery chance. On the other hand, age may also be linked to other factors, especially the IUI attempt rank. It is logical that, with more IUI attempts, the age advances. For this reason, Aydin et al. [68] could find no significant effect of female age on the clinical pregnancy rate in the first IUI cycle. Indeed, the rank attempt is determinant for IUI success. In our study, pregnancy rates and live births decreased significantly with the rank of insemination (*p* = 0.03 and *p* < 0.01, resp.) from rank 4 for both parameters (*p* = 0.02, see Table 6). Hendin et al. [67] and Merviel et al. [30] obtained 97% and 80%, respectively, of clinical pregnancies in their first three attempts. Plosker et al. [69] advocated a passage in IVF after three failed cycles of IUI. However, Soria et al. [15] demonstrated that from the fourth IUI cycle clinical pregnancy is negatively affected, which confirms our results.

However, Blasco et al. [62] proved that the number of previous IUI cycles of the patient did not show a positive association with the cycle outcome in any of the developing steps of the models. In our study, IUI attempt rank did not have a clear correlation with clinical outcomes in different COS groups, but it did show a negative correlation with live-birth rates for patients with PCOS, unexplained infertility and male factor (Tables 3 and 5). This could be explained by the evidence of severity of infertility type throughout time with an accumulation of IUI attempt failures, while IUI as a simple technique is less efficient than other ART techniques in achieving a clinical pregnancy. Particularly for infertile couples with male factor, the sperm quality becomes the determinant for IUI success [11, 70, 71], which was shown in our findings with a positive correlation of sperm concentration (Table 5). It would be difficult to determine a universal threshold for sperm concentration, and each centre should define a threshold for its population and laboratory [72]. Nevertheless, Belaisch-Allart et al. [73] and Sakhel et al. [74] determined a sperm concentration cut-off at 10 × 10^6^/mL and 5 × 10^6^/mL, respectively. Indeed, the impact of semen quality was weak in our study, except for concentrations <5 × 10^6^/mL, which remains nonsignificant due to small numbers of patients (8% of included population) (Table 6)

Sperm motility also appeared as a key factor in the study of Merviel et al. [30], where the pregnancy rate declined from 41% to 19% when the sperm motility was less than 70%. In our multivariable analysis with a sperm motility cut-off at 40%, we did not find any significant correlation with IUI clinical outcomes even with a large population size. This observation is reported also by Stone et al. [75].

However, the TMS cut-off at 1 × 10^6^, which was present in 21% of the included infertile patients, was a significant predictor of IUI clinical pregnancy (Table 6). This finding was confirmed by two studies [9, 10] while others determined a higher threshold of TMS at 2 × 10^6^ [68]; 3 × 10^6^ [62, 76]; 5 × 10^6^ [11, 77]; 10 × 10^6^ [63, 78]. Indeed, the IUI clinical outcomes were improved with higher TMS, from 3.6 × 10^6^ to 12 × 10^6^ [38]. Furthermore, regarding the sperm parameters, TMS was found to be an independent factor for clinical pregnancy after IUI in accordance with many authors [28, 63, 74, 77, 79–81]. However, Ozkan et al. [82] found just a minimal influence of TMS on the IUI success after washing.

Nevertheless, TMS is a key factor for choosing IUI treatment or IVF, although a TMS threshold value of 5 × 10^6^ to 10 × 10^6^ has been reported as the criterion for undergoing IVF. Nevertheless, other sperm parameters could be better predictors of sperm morphology [58]. Although the predictive weakness of conventional sperm parameters for ART clinical outcomes has been demonstrated, sperm genome decay tests [83] could become a strong diagnostic tool to achieve clinical pregnancy for infertile couples undergoing homologous IUI.

Other predictive factors for success have been found in some studies, such as duration of infertility, body mass index [15, 60, 82, 84, 85], and smoking [37], which were not regularly noted in our records and, therefore, could not be analysed.

## 5. Conclusion

This study, is in concordance with our preliminary work [86] and demonstrate that there is no significant difference in clinical outcomes between different COS protocols rFSH, uFSH, or hMG and infertility types, even after taking into account the usual prognostic factors, including the female's age, the IUI attempt rank, and the sperm quality. However, unexplained infertility had a significant impact on IUI success, which revealed the need to look for more efficient ART strategies. Furthermore, since the fourth IUI attempt or with the female aged over 40 years, clinical pregnancy declined in IUI. Regarding the sperm quality, TMS with a threshold of 5 × 10^6^ seemed a good predictor for IUI success. Indeed, over the obtained cut-off of the chosen indicators, other ART techniques might be more favourable for IVF live-birth rates.

For infertile patients with male factor, sperm concentration was a determinant to achieve pregnancy, which necessitated some additional tests, such as sperm genome decay tests, before undergoing IUI and reviewing the couple's etiological factors for antioxidant prescriptions. Finally, every decision must be individualized to each couple's profile taking into account factors involved in the success of IUI.

## Figures and Tables

**Table 1:** Baseline characteristics of couples included in the study.

Characteristics	Total number	M ± SD/rate (%)
IUI cycle number	1251	-
COS		
rFSH/Gonal F	362	29%
rFSH/Puregon	538	43%
uFSH/Fostimon	210	17%
hMG/Menopur	141	11%
Infertility type		
Cervical	276	22%
Dysovulation	91	7%
PCOS	205	16%
Endometriosis	35	3%
Tubal factor	44	3%
Male factor	153	12%
Unexplained infertility	447	36%
Women's age (years)	1251	31.6 ± 4.3
IUI attempt rank	1251	2.2 ± 1.3
Semen quality		
Sperm concentration (×10^6^/ml)	1251	76.0 ± 73.7
Sperm total motility (%)	1251	24 ± 0.12
TMS (×10^6^)	1251	9.4 ± 6.2
Cycle outcome		
Clinical pregnancy	166	13.3%
Live birth	142	11.4%

Results are expressed as *n* and % or *n* and mean (M) ± standard deviation (SD) according to the variable nature; IUI: intrauterine insemination; COS: controlled ovarian stimulation; PCOS: polycystic ovary syndrome; TMS: total motile sperm count.

**Table 2:** Comparison between the 4 groups of ovarian stimulation.

Characteristics	rFSH/Gonal F (*n* = 362)	rFSH/Puregon (*n* = 538)	uFSH/Fostimon (*n* = 210)	hMG/Menopur (*n* = 141)	*p* value
Women's age (years)	31.5 ± 4.2	31.6 ± 4.2	31.3 ± 4.5	32.4 ± 4.0	0.10
IUI attempt rank	2.22 ± 1.31	2.20 ± 1.33	1.97 ± 1.09	2.27 ± 1.25	0.15
Sperm quality					
Sperm concentration (×10^6^/ml)	71.4 ± 64.5	76.4 ± 67.1	79.4 ± 76.2	81.6 ± 108.3	0.75
Sperm motility (%)	25 ± 12	24 ± 11	25 ± 12	22 ± 13	0.06
TMS (×10^6^)	9.3 ± 5.7	9.5 ± 6.1	9.3 ± 6.6	9.4 ± 7.3	0.62
Clinical outcomes					
Clinical pregnancy rate (*n*; %)	46; 12.7	72;13.4	28; 13.3	20; 14.2	0.98
Live birth rate (*n*; % cycle)	42; 11.6	62; 11.5	24; 11.4	19; 13.5	0.99

Results are expressed as *n* and %, or *n*, and mean (M) ± standard deviation (SD) according to variable nature; IUI: intrauterine insemination; TMS: total motile sperm count.

**Table 3:** Correlation between of couple's parameters and clinical outcomes relatively to used COS for IUI.

Couple's parameters	rFSH/Gonal F (*n* = 362)		rFSH/Puregon (*n* = 538)		uFSH/Fostimon (*n* = 210)		hMG/Menopur (*n* = 141)	
	CP	LB	CP	LB	CP	LB	CP	LB
Women's age (years)	−0.03	−0.03	−0.02	−0.04	−0.07	−0.05	−0.01	−0.01
IUI attempt rank	−**0.17 (s)**	−**0.22 (s)**	−**0.16 (s)**	−**0.16 (s)**	−**0.18 (s)**	−**0.23 (s)**	−**0.21 (s)**	−**0.22 (s)**
Sperm quality								
Sperm concentration (×10^6^/ml)	0.01	0.01	0.08	0.06	0.03	0.06	0.07	0.08
Sperm motility (%)	0.09	**0.16 (s)**	0.03	**0.17 (s)**	0.08	0.09	0.02	0.02
TMS (×10^6^)	0.05	0.13	0.03	0.09	0.08	0.05	0.07	0.04

Results are expressed as *r* values representing the correlation coefficient calculated for each parameter relative to each clinical outcome. *r* were considered significant (s) for *p* < 0.05; IUI: intrauterine insemination; TMS: total motile sperm count; CP: clinical pregnancy; LB: live birth.

**Table 4:** Comparison between the 7 groups of infertility type.

Couple's parameters	Cervical (*n* = 276)	Dysovulation (*n* = 91)	PCOS (*n* = 205)	Endometriosis (*n* = 35)	Tubal factor (*n* = 44)	Male factor (*n* = 153)	Unexplained infertility (*n* = 447)	*p* value
Women's age (years)	31.8 ± 4.1	31.7 ± 3.8	30.1 ± 3.6	30.5 ± 3.8	31.1 ± 5.1	31.6 ± 3.8	32.3 ± 4.6	0.11
IUI attempt rank	2.36 ± 1.38	2.22 ± 1.25	2.11 ± 1.21	1.86 ± 0.94	1.73 ± 0.85	2.22 ± 1.33	2.14 ± 1.28	0.16
Sperm quality								
Sperm concentration (×10^6^/ml)	87.1 ± 97.9	77.0 ± 82.9	76.9 ± 69.8	54.4 ± 37.9	111 ± 81.6	46.4 ± 46.1	76.9 ± 63.6	0.11
Sperm motility (%)	25 ± 14	22 ± 12	24 ± 12	28 ± 10	28 ± 14	17 ± 10	26 ± 12	0.10
TMS (×10^6^)	9.4 ± 6.1	10.4 ± 8.3	9.5 ± 5.6	9.7 ± 6.8	11.6 ± 8.4	6.4 ± 5.3	10.0 ± 5.8	0.09
Clinical outcomes								
Clinical pregnancy rate (*n*; %)	40; 14.5	16; 17.6	31; 15.1	3; 8.6	5; 11.4	23; 15.0	48; 10.7	0.41
Live birth rate (*n*; %)	36; 13.0	14; 15.4	25; 12.2	3; 8.6	4; 9.1	21; 13.7	44; 9.8	0.76

Results are expressed as *n*, *n* (%), or mean (M) ± standard deviation (SD). A difference was considered significant (s) when *p* < 0.05; ns: not significant. The power 1 − *β* was calculated based on the difference of mean values between groups with *α* = 0.05. The power value was considered important when above 80%. The power mean value of this part of study is 61%; IUI: intrauterine insemination; PCOS: polycystic ovary syndrome; TMS: total motile sperm count.

**Table 5:** Correlation between couple's parameters and clinical outcomes relatively to infertility type.

Characteristic	Cervical (*n* = 276)		Dysovulation (*n* = 91)		PCOS (*n* = 205)		Endometriosis (*n* = 35)		Tubal factor (*n* = 44)		Male factor (*n* = 153)		Unexplained infertility (*n* = 447)	
	CP	LB	CP	LB	CP	LB	CP	LB	CP	LB	CP	LB	CP	LB
Women's age (years)	−0.01	−**0.32 (s)**	−0.03	−0.12	−0.05	−0.09	−0.33	−0.22	−0.02	−0.35	−0.02	−0.19	−0.02	−0.15
IUI attempt rank	−0.06	−0.19	−0.05	−0.08	−0.03	−**0.37 (s)**	−0.17	−0.18	−0.12	−0.61	−0.16	−**0.18 (s)**	−0.03	−**0.33 (s)**
Sperm quality														
Sperm concentration (×10^6^/ml)	0.03	0.18	0.20	0.37	0.04	0.01	0.02	0.02	0.19	0.35	**0.22 (s)**	**0.24 (s)**	0.02	0.01
Sperm motility (%)	0.01	0.26	0.11	0.11	0.03	0.20	0.09	0.08	0.01	0.36	0.01	0.29	0.05	0.04
TMS (×10^6^)	0.02	0.17	**0.29 (s)**	0.16	0.01	0.09	0.03	0.03	0.09	0.35	0.01	0.07	0.05	0.11

Results are expressed as *r* values representing the correlation coefficient calculated for each parameter relative to each clinical outcome. *r* were considered significant (s) for *p* < 0.05; IUI: intrauterine insemination; TMS: total motile sperm count; CP: clinical pregnancy; LB: live birth.

**Table 6:** Multivariate analysis of IUI success factors.

IUI success factors	Clinical pregnancy			Live birth		
	OR	95% CI	*p* value	OR	95% CI	*p* value
COS (hMG versus FSH)	1.11	0.66–1.86	0.70	1.28	0.75–2.17	0.37
Infertility type						
Cervical	1.00	—	—	1.00	—	—
Dysovulation	1.14	0.62–2.10	0.69	1.07	0.55–2.07	0.85
PCOS	0.91	0.56–1.48	0.70	0.82	0.48–1.39	0.46
Endometriosis	0.38	0.13–1.10	0.07	0.46	0.16–1.35	0.16
Tubal factor	0.61	0.23–1.64	0.32	0.59	0.20–1.73	0.33
Male factor	0.91	0.56–1.48	0.69	1.00	0.60–1.67	1.00
Unexplained infertility	**0.63**	**0.40**–**1.00**	**0.047 (s)**	0.68	0.42–1.11	0.13
Female age (≥40 years versus ≤39)	**0.17**	**0.02**–**1.28**	**0.09 (s)**	0.19	0.03–1.40	0.10
IUI attempt rank (≥4 versus ≤3)	**0.51**	**0.29**–**0.89**	**0.02 (s)**	**0.47**	**0.25**–**0.88**	**0.02 (s)**
Sperm quality						
Sperm concentration (<15 × 10^6^/ml versus >15)	0.81	0.40–1.65	0.56	0.79	0.37–1.66	0.52
Sperm motility (≥40 % versus ≤39)	1.43	0.86–2.39	0.17	1.39	0.80–2.40	0.24
TMS (≥5 × 10^6^ versus <5)	**1.02**	**1.00**–**1.04**	**0.047 (s)**	1.05	0.87–1.28	0.60

Multivariate analysis was performed by logistic models; IUI: intrauterine insemination; COS: controlled ovarian stimulation; PCOS: polycystic ovary syndrome; TMS: total motile sperm count.

## Abbreviations

ART: Assisted reproductive technologies
COS: Controlled ovarian stimulation
IUI: Intrauterine insemination
PCOS: Polycystic ovaries syndrome
TMS: Total motile sperm.
